# Association of the *MUC5B* promoter polymorphism with idiopathic pulmonary fibrosis in a lebanese cohort

**DOI:** 10.3389/fgene.2025.1544864

**Published:** 2025-06-24

**Authors:** Antoine Mouawad, Eliane Chouery, Alain Chebly, Nabiha Salem, Sandra Corbani, Maissa Safieddine, Georges Dabar

**Affiliations:** ^1^ Faculty of Medicine, Saint Joseph University of Beirut, Beirut, Lebanon; ^2^ Center Jacques Loiselet for Medical Genetics and Genomics (CGGM), Faculty of Medicine, Saint Joseph University of Beirut, Beirut, Lebanon; ^3^ Centre de Recherche Clinique, Faculty of Medicine, Saint Joseph University of Beirut, Beirut, Lebanon; ^4^ Department of Pulmonology and Critical Care, Hotel Dieu de France Hospital, Beirut, Lebanon

**Keywords:** idiopathic pulmonary fibrosis, risk factor, MUC5B, rs35705950, promoter polymorphism, single nucleotide polymorphism, Lebanon

## Abstract

**Background and objective:**

Idiopathic Pulmonary Fibrosis (IPF) is an interstitial lung disease that causes irreversible alterations in the architecture of the lung parenchyma, leading to impaired ventilation. Both environmental factors and genetic predisposition play significant roles in the development of IPF. A single nucleotide polymorphism (SNP) (rs35705950) within the promoter of the mucin 5B gene (*MUC5B)* has been reported to be associated with the disease; however, no data is available from Lebanon or the Middle East. This study aims to identify the frequency of the *MUC5B* promoter variant among a cohort of Lebanese IPF patients, compare it to the general population and assess its association with the risk of developing the disease.

**Methods:**

A total of 55 patients diagnosed with IPF, according to the ATS/ERS criteria, and 94 healthy controls were included in the study. DNA samples were extracted and genotyped for the *MUC5B* promoter polymorphism by Sanger sequencing. Descriptive statistics were performed on clinical characteristics. Pearson’s chi-squared and T-student tests were performed to determine statistical significance. Odds ratios quantified genetic variant associations with IPF.

**Results:**

The *MUC5B* SNP rs35705950 was significantly more frequent in IPF patients compared to the control group, in both heterozygous and homozygous forms. Additionally, a significant association was found between the variant and susceptibility to IPF.

**Conclusion:**

This study shows that the *MUC5B* polymorphism rs35705950 is significantly more frequent in the Lebanese IPF population compared to the control group and is associated with an increased risk of developing IPF.

## Introduction

Idiopathic pulmonary fibrosis (IPF, OMIM #178500) is an interstitial lung disease characterized by irreversible alterations in the architecture of the lung parenchyma, ultimately leading to impaired oxygenation and ventilation. IPF is a progressive disease, bearing a poor prognosis with a median survival of 2–5 years (Frankel and Schwarz, 2009). A deep understanding of its underlying factors and pathophysiology is essential to enable a better management of the disease and improve its outcomes. However, this goal remains unachieved, as no clear mechanism has yet been defined, nor has any single factor been identified as a direct cause involved in the onset of this disease.

Nonetheless, several environmental factors, such as exposure to metal dust, wood dust and pesticides, as well as occupational environments like the agricultural sector and wood industry, have been shown to significantly increase the risk of developing IPF (Park et al., 2021). Additionally, smoking as well as smoking exposure, known risk factors in many pulmonary diseases, are also associated with an increased risk of IPF. A dose-response relationship has been suggested between pack-years of smoking and IPF risk; though the precise involved mechanism remains unclear (Bellou et al., 2021). Another reported risk factor for IPF includes chronic viral infections, such as EBV, CMV and HHV-7/8, which have been implicated in increasing susceptibility to IPF (Sheng et al., 2020).

Over the past decade, genetic predisposition to IPF has been also extensively investigated, with several genetic variants reported as being involved in IPF pathogenesis. Among them are variants in the telomerase reverse transcriptase (*TERT*), desmoplakin (*DSP*) and toll-interacting protein (*TOLLIP*) genes (Singh et al., 2024; Borie et al., 2022; Bonella et al., 2021; Guzmán-Vargas et al., 2021; Oldham et al., 2015). Similarly, a single nucleotide polymorphism (SNP), rs35705950, in the promoter region of the mucin 5B (*MUC5B*, OMIM: 600770) gene, located 3 kb upstream of the transcription start site has been extensively reported to be associated with IPF (Seibold et al., 2011). The MUC5B protein, secreted by submucosal glands and epithelial secretory club cells (Okuda et al., 2019), plays a major role in airway defense. Elevated levels of MUC5B are believed to compromise airway defense mechanisms, potentially contributing to lung injury from inhaled substances and smoke (Hancock et al., 2018).

Although the exact role of the rs35705950 variant in IPF pathogenesis is not fully understood, multiples studies have demonstrated that the presence of the minor T allele (in both heterozygous and homozygous states) correlates with increased MUC5B expression in the lung parenchyma. Notably, Seibold et al. reported that, in a Western population of European ancestry, 38% of IPF patients present the minor T allele of the *MUC5B* promoter, compared to 9% of controls (Seibold et al., 2011). This association has been confirmed in populations from the United States (Seibold et al., 2011; Dressen et al., 2018; Helling et al., 2017), Mexico (Peljto et al., 2015), and Europe (Bonella et al., 2021; Allen et al., 2017; Borie et al., 2013), establishing the *MUC5B* promoter polymorphism as the strongest genetic risk factor for IPF. However, in East Asian populations, including those from Japan and China (Horimasu et al., 2015; Deng et al., 2018), the association is weaker and sometimes even absent (Peljto et al., 2015). All this shows the high variability in the minor allele frequency of this variant, depending on the population and region studied.

To our knowledge, no data on the association of the *MUC5B* promoter polymorphism and IPF is available from the Middle East region. This study represents the first report on its association with IPF in the Lebanese population, aiming to assess the association of the *MUC5B* promoter polymorphism and IPF in Lebanon.

## Materials and methods

### Participant recruitment

Between January 2017 and December 2019, 55 Lebanese patients with IPF were recruited from Hotel Dieu de France (HDF) Hospital in Beirut (Lebanon). After excluding secondary causes (connective tissue disease, drugs or environmental exposures), diagnosis of IPF was established based on clinical (pulmonary function tests) and imaging findings (CT scan with or without histopathological confirmation) in accordance with the criteria of the American Thoracic Society (ATS) and European Respiratory Society (ERS) (Raghu et al., 2018). Thoracic imaging was reviewed by an independent radiologist who then graded the computed tomography of the chest as UIP or probable UIP (ATS guidelines 2018). Additionally, 94 Lebanese healthy controls were included from our healthy patients database. This study was approved by the Ethics Committee at HDF Hospital and Saint-Joseph University (USJ) of Beirut (N-716). It was conducted in accordance with the ethical standards established by the Helsinki Declaration of 1975. All participants provided written informed consent to participate in this study.

### Sample size calculation

The study investigated the association between the *MUC5B* promoter polymorphism and IPF. The required sample size was estimated based on previously published studies, assuming a T allele prevalence of 10% in controls, 30% in IPF, and an odds ratio of 4. Using G*Power software, a total of 131 participants (44 cases and 87 controls) was calculated to achieve 80% power with a significance level alpha of 0.05 and a 1:2 case-to-control ratio, reflecting the rarity of IPF in Lebanon.

### DNA preparation and SNP genotyping

#### DNA extraction

DNA was extracted for all patients from dried blood spots on Guthrie cards using the QIAamp DNA^®^ Mini Kit (Qiagen). As for the controls, DNA was extracted directly from peripheral blood using the same DNA extraction kit.

#### DNA sequencing

To genotype the rs35705950 polymorphism in the study participants, primers were designed, using Primer 3 (https://primer3.ut.ee/) to amplify a segment of the 5’UTR region of the *MUC5B* gene (NM_002458.3). The reference DNA sequence was obtained from UCSC (https://genome.ucsc.edu/) based on the hg19 build (GRCh37). Primers specificity was checked using the online tool BLAST. The primers sequences are: 5’-gcc​aga​atg​agg​gac​agt​ga-3’ (forward) and 5’-acg​tca​agg​cca​cag​cta​tt-3’ (reverse). Polymerase chain reactions (PCR) were performed using the Qiagen multiplex PCR kit (Qiagen). The amplification conditions were: 95°C for 15 min, followed by 35 cycles of: 95°C for 30 s, 59°C for 60 s, and 72°C for 30 s, with a post-cycling final extension of 15 min at 72°C. The amplified fragments were purified using GenElute PCR clean-up kit (SIGMA-ALDRICH™). Sequencing reactions were prepared using the Big Dye Terminator v1.1 Cycle sequencing kit (Thermo Fisher Scientific) under standard conditions. They were purified using the Sephadex G50 (Amersham Pharmacia Biotech) and then loaded into an ABI 3500 Genetic Analyzer (Thermo Fisher Scientific) for capillary electrophoresis. Electropherograms were generated with Sequence Analysis Software version 5.4 (Thermo Fisher Scientific) and then compared to the reference sequence using ChromasPro v2.1.9 (Technelysium).

### Statistical analyses

Descriptive statistics on the clinical characteristics of the patients included in this study was performed. Patient characteristics are presented as mean and standard deviation (SD) in the case of continuous data, and absolute and relative frequencies in the case of categorical data. Pearson’s chi-squared and T-student tests were performed to determine statistical significance. Additionally, to quantify associations between genetic variants and IPF, we calculated odds ratios (ORs) along with their 95% confidence intervals (CIs) for different genotypes. A p-value of <0.05 was considered statistically significant. The analyses were performed using R statistical software version 4.1.2. In addition, we performed a logistic regression under an additive genetic model to assess the association between the number of T alleles and IPF, adjusting for age and sex as covariates.

## Results

### Population characteristics

The characteristics of the 149 participants (patients and controls) are summarized in Table 1. The mean age is 78.73 years (±10.19) in the IPF group and 70.37 (±10.08) in the control group. Sex distribution is similar in both groups, with 67.3% and 67% of males in the IPF and control groups, respectively.

**TABLE 1:** Characteristics and demographics of the IPF patients and controls included in this study.

	IPF	Controls
N	55	94
Age: mean (SD)	78.73 (10.19)	70.37 (10.08)
Sex: N of males (%)	36 (67.3)	63 (67.0)

### *MUC5B* rs35705950 genotyping

Obtained genotyping results of the patients with IPF and the controls are summarized in Table 2. The *MUC5B* promoter variant rs35705950 was found at both heterozygous (GT) and homozygous (TT) states in IPF patients, with an overall frequency of 70%, compared to 36.2% in controls (p < 0.001). When stratifying by genotype, IPF patients exhibited a significantly higher frequency of the *MUC5B* rs35705950 variant at homozygous state compared to controls [20% vs. 4.3% (p = 0.005)]. Similarly, the heterozygous form was more prevalent in IPF patients than in controls [50.9% vs. 31.9% (p = 0.034)]. The minor allele frequency (MAF) among the IPF group was 45.5% compared to 20.2% in the control group (p < 0.001).

**TABLE 2:** MUC5B rs35705950 frequency in IPF patients and controls, and its association with IPF.

	IPF N (%)	Controls N (%)	p-value	Or (95% CI)
	55 (100%)	94 (100%)		
GG	16 (29.1)	60 (63.8)	<0.001	
GT	28 (50.9)	30 (31.9)	0.034	3.50 (1.67–7.58)
TT	11 (20.0)	4 (4.3)	0.005	10.31 (3.09–41.36)
MAF (T)	45.5	20.2	<0.001	

Additionally, we observed a significant association between the presence of the variant and IPF risk. Indeed, the heterozygous form (GT) showed an odds ratio (OR) of 3.50 (95% CI: 1.67–7.58), and the homozygous form (TT) an OR of 10.31 (95% CI: 3.09–41.36); both representing significant risk factors for IPF compared to individuals carrying the GG genotype. As for the logistic regression analysis, it showed that each additional T allele significantly increased the odds of IPF (OR = 2.96, 95% CI: 1.67–5.46, p < 0.001). Age was also a significant predictor (OR = 1.08, 95% CI: 1.04–1.13, p < 0.001), which indicates an 8% increase in the risk of developing IPF with each additional year of age, while sex was not associated with IPF (OR = 0.86, 95% CI: 0.39–1.93, p = 0.715).

## Discussion

In this study, we report for the first time data from Lebanon on the *MUC5B* promoter variant rs35705950 and its association with IPF in Lebanese patients. The patient and control groups were comparable, with mean ages of 78 and 70 years respectively, and a similar sex distribution of approximately 67% of males in each group. This comparability is relevant, as the average age of IPF diagnosis varies between 50 and 70 years of age, with most patients developing symptoms after 60 (Castriotta et al., 2010). Similarly, the male-to-female ratio for IPF generally varies between 1.6:1 to 2:1 (Sesé et al., 2021).

Our findings demonstrate a significantly higher frequency of the rs35705950 minor allele (T) in the *MUC5B* gene among Lebanese IPF patients compared to controls. This suggests that the T allele, both at heterozygous or homozygous state, is associated with a higher risk for developing IPF.

Hardy-Weinberg equilibrium (HWE) was assessed in both the control and IPF groups. In the control group, statistical studies revealed that the p-value (0.993) is substantially higher than the significance level (0.05), therefore we cannot reject the null hypothesis. This suggests that the observed genotype frequencies are not statistically different from those expected under HWE. As a result, the control group is in HWE, and there is no indication of major genotyping errors or biases in the sample. However, in the IPF group, the null hypothesis of HWE is rejected, showing that the observed genotype frequencies are considerably different from the expected frequencies, implying that the IPF population is not in HWE. All these results support the hypothesis that the *MUC5B* promoter SNP might influence IPF disease risk.

MUC5B, like other mucins and secreted proteins in the respiratory airways, plays an important role in airway defense. In healthy lungs, MUC5B is expressed in the cytoplasm of goblet cells. However, in IPF patients, MUC5B is found to be accumulated in areas of microscopic honeycombing (Seibold et al., 2011). Previous studies, such as that by Seibold et al. established a correlation between the T allele of rs35705950 and overexpression of *MUC5B* mRNA in the lung parenchyma (Seibold et al., 2011). Our results combined with findings from similar studies in other populations, reinforce the role of the *MUC5B* promoter polymorphism as a risk for IPF development. Although the mechanism behind this association remains largely unknown, a recent study on mice highlighted the role of endoplasmic reticulum (ER) stress that could be associated with this variant and identified potential therapeutic agents targeting this pathway (Dobrinskikh et al., 2023).

The association between the *MUC5B* promoter polymorphism and IPF varies across populations (Table 3). In the United States, Seibold et al. found that the MAF for the *MUC5B* polymorphism was 38% among IPF patients compared to 9% in controls (Seibold et al., 2011). Other studies in the United States have confirmed this association with reports indicating that 51% of IPF patients have the T allele versus 23% of controls (Helling et al., 2017). Subsequent studies have consistently reported similar genotype distributions as per the study of Seibold et al., with percentages varying between 33% and 54% for IPF heterozygous patients and 3%–7% being homozygous (Ley et al., 2017; Wei et al., 2014; Zhang et al., 2011). Our results align with these findings, although our cohort showed a higher proportion of homozygous cases.

**TABLE 3:** Worldwide data reporting on the association between rs35705950 and IPF.

Authors	Country	MAF %		GT %		TT %		References
		IPF	Controls	IPF	Controls	IPF	Controls	
Yusuf et al.	Egypt	N/A	N/A	50	15	11.9	5	Yusuf et al. (2020)
Seibold et al.	United States	37.5	9.1	N/A	N/A	N/A	N/A	Seibold et al. (2011)
Helling et al.	United States	N/A	N/A	43	23	7	0	Helling et al. (2017)
Ley et al.	United States (UCSF)	33.3	10.7	38	N/A	3	N/A	Ley et al. (2017)
Ley et al.	United States (UTSW)	32.0	10.7	44	N/A	8	N/A	Ley et al. (2017)
Wei et al.	United States	30	12	52	20	3.5	1.6	Wei et al. (2014)
Zhang et al.	United States	34.3	11.1	54.6	7	19.2	1.5	Zhang et al. (2011)
Stock et al.	United Kingdom (Caucasians)	36	10	N/A	N/A	N/A	N/A	Stock et al. (2013)
Bonella et al.	Germany (Caucasians)	35	9	56	14	6	2	Bonella et al. (2021)
Borie et al.	France (Caucasians)	41.9	10.81	53.57	18.73	11.9	1.45	Borie et al. (2013)
Van der Vis et al.	Netherlands	27	9	45	17	4	0	Vis et al. (2022)
Jiang et al.	China (Chinese Han)	19.3	11	N/A	N/A	N/A	N/A	Jiang et al. (2015)
Wang et al.	China (East Asian)	3.33	0.79	6.67	1.58	N/A	N/A	Wang et al. (2014)
Horimasu et al.	Japan	3.4	0.8	6.8	1.6	0	0	Horimasu et al. (2015)
Horimasu et al.	Germany	33.1	4.3	43.6	9.6	11.3	0	Horimasu et al. (2015)
Peljto et al.	Mexico	25	N/A	N/A	N/A	N/A	N/A	Peljto et al. (2015)
Peljto et al.	Korea	1	N/A	N/A	N/A	N/A	N/A	Peljto et al. (2015)
Mouawad et al.	Lebanon	45.5	20.2	50.9	31.9	20	4.3	Current study

In European studies, including those conducted in the UK, France, Germany, and the Netherlands, the *MUC5B* polymorphism has also been significantly associated with IPF, with MAF varying from 27% to 42% among IPF patients, compared to 9%–10% in the respective control groups (Bonella et al., 2021; Allen et al., 2017; Borie et al., 2013; Stock et al., 2013; Vis et al., 2022; Kishore et al., 2016). Interestingly, these studies demonstrated that there is no association between this variant and the pulmonary fibrosis present in conditions like systemic sclerosis and sarcoidosis, suggesting a specific role for the studied MUC5B polymorphism in IPF pathogenesis. However, studies interested in other autoimmune diseases such as rheumatoid arthritis have shown an association with the *MUC5B* promoter variant. In fact, a study conducted in 2018 showed a positive association between this variant and the diagnosis of RA-related ILD (Juge et al., 2018). This highlights the poor understanding we have of the mechanism behind this variant and its association with ILD.

On another hand, our findings most closely resemble to data from a French cohort with a MAF of 41.9% in the IPF group versus 10.8% in the control group, and a similar genotype distribution: 53.57% heterozygous (GT), and 11.9% homozygous (TT) in IPF patients, compared to 18.73% and 1.45% respectively in controls (Borie et al., 2013). Lastly, a recent study in Egypt also confirmed the association between the rs35705950 variant and IPF, with similar genotype distributions (50% of heterozygous and 12% of homozygous patients compared to 15% and 5%, respectively in controls) (Yusuf et al., 2020).

In contrast, studies in Eastern Asia present a less consistent association between the *MUC5B* promoter polymorphism and IPF. Indeed, a study conducted in China found that MAF was 19.3% in the IPF group compared to 11% in the control group, although this association was found to be significant, it suggests a lower risk in this population compared to those in Europe and North America (Jiang et al., 2015). Similarly, but in a larger Chinese cohort of 165 IPF patients, none of the patients were homozygous for the rs35705950, and only 6.67% were heterozygous compared to 1.58% in controls suggesting once again a lower association in East Asian populations than in Western ones (Wang et al., 2014).

A direct comparison between a Japanese and a German cohort revealed that the T allele frequency was significantly higher in a German IPF group compared to the Japanese IPF group. Horimasu et al. also found that it was significantly lower in both Japanese IPF and control groups compared to German counterparts, concluding that this association is present in the Japanese population, but not as strong as in Western populations (Horimasu et al., 2015). Another study comparing a Mexican and a Korean cohort found that the rs35705950 had no significant effect on the Korean population (Peljto et al., 2015).

Furthermore, when comparing our cohort to publicly available data from the gnomAD database (v4.1.0), we found that the MAF across different populations was consistent with our results. In fact, the second highest MAF reported was in the Middle Eastern population (0.1327). Some populations with MAF values close to ours include the Amish (MAF = 0.1458) and European non-Finnish (MAF = 0.1103), while East Asian populations present much lower MAF (0.006952). This would explain the low association found between the rs35705950 and IPF development in the East Asian populations.

In conclusion, the association between the *MUC5B* SNP and IPF susceptibility is not constant across all populations; the strongest association was found in Europe and United States populations. Our findings are more in line with the western studies, and our cohort was found to be the closest to European one, in particular to the French population.

This study has certain limitations, primarily the relatively small sample size. However, when compared to other studies and considering Lebanon’s population size, our findings still underscore the *MUC5B* promoter polymorphism as a significant risk factor for IPF in Lebanese patients. These findings are important however future research should investigate the link between the rs35705950 SNP and disease severity, progression, radiologic features and survival among different genotypes in IPF patients. This would enhance our understanding of the variant’s role in IPF onset, progression, and prognosis.

Overall, our results provide the first data on the *MUC5B* promoter polymorphism and its role as a major risk factor for IPF in Lebanon. These results form a foundation for further studies on this variant and its impact on IPF severity and prognosis.

## Conclusion

For the first time in Lebanon and the Middle East, we report on the *MUC5B* promoter variant rs35705950 as a major risk factor for the development of IPF. Our findings align with studies from Europe and the United States. Larger global studies are needed to better understand the role of the rs35705950 variant in IPF and its correlation to clinical, functional and radiological features of the disease.

## Data Availability

The original contributions presented in the study are included in the article/supplementary material, further inquiries can be directed to the corresponding authors.
